# Multiple Chronic Condition Combinations, Affect, and Memory Among Black and White Older Adults

**DOI:** 10.1093/geroni/igab046.704

**Published:** 2021-12-17

**Authors:** Aaron Ogletree, Steph Cooke, Benjamin Katz

**Affiliations:** 1 National Institute on Minority Health and Health Disparities, Washington, District of Columbia, United States; 2 Virginia Tech, Blacksburg, Virginia, United States

## Abstract

Research demonstrates the adverse effects of coexisting multiple chronic conditions (MCCs) on older adults’ health and wellbeing. While most research relies on total counts of chronic conditions, little work explores how specific MCC combinations may have compounding effects on depression and memory. Furthermore, no published research explores differences in the prevalence and correlates of MCC combinations between Black and White older adults. The current study assesses within- and between-group heterogeneity in the prevalence and correlates of MCC combinations to advance health equity research. We utilize a sample of 16,757 Black and White older adults drawn from the 2014 wave of the Health and Retirement Study. Respondents were categorized into one of 32 MCC combination groups. Depressive symptoms and self-rated memory were calculated separately for Black and White respondents across each of the 32 groups. Chi-square tests, t-tests, and ANCOVAs were used to compare differences. Black and White respondents differed significantly in the prevalence of 14 out of 32 MCC combinations. Within-group differences were found such that 45% of Black respondents experiencing only Lung Disease met criteria for clinical depression; this rate is similar to Black respondents experiencing Diabetes + Heart Condition + Hypertension + Lung Disease (44.5%). Between-group differences revealed that Black respondents experiencing Arthritis + Diabetes + Hypertension had worse self-rated memory than White counterparts (MB = 3.24, MW = 3.13; two sample t[1139]= -2.04, p < .05; Cohen’s d = 0.13). Additional findings are presented, and theoretical and practical implications for this work are discussed.

